# DeepPN: a deep parallel neural network based on convolutional neural network and graph convolutional network for predicting RNA-protein binding sites

**DOI:** 10.1186/s12859-022-04798-5

**Published:** 2022-06-29

**Authors:** Jidong Zhang, Bo Liu, Zhihan Wang, Klaus Lehnert, Mark Gahegan

**Affiliations:** 1 Faculty of Information Technology, Beijing University of Technology, Beijing, 100124 China; 2 School of Mathematical and Computational Sciences, Massey University, Palmerston North, 4472 New Zealand; 3 School of Biological Sciences, University of Auckland, Auckland, 1142 New Zealand; 4 School of Computer Science, the University of Auckland, Auckland, 1010 New Zealand

**Keywords:** Bioinformatics, RNA-binding protein, Convolutional neural network, Graph convolution network

## Abstract

**Background:**

Addressing the laborious nature of traditional biological experiments by using an efficient computational approach to analyze RNA-binding proteins (RBPs) binding sites has always been a challenging task. RBPs play a vital role in post-transcriptional control. Identification of RBPs binding sites is a key step for the anatomy of the essential mechanism of gene regulation by controlling splicing, stability, localization and translation. Traditional methods for detecting RBPs binding sites are time-consuming and computationally-intensive. Recently, the computational method has been incorporated in researches of RBPs. Nevertheless, lots of them not only rely on the sequence data of RNA but also need additional data, for example the secondary structural data of RNA, to improve the performance of prediction, which needs the pre-work to prepare the learnable representation of structural data.

**Results:**

To reduce the dependency of those pre-work, in this paper, we introduce DeepPN, a deep parallel neural network that is constructed with a convolutional neural network (CNN) and graph convolutional network (GCN) for detecting RBPs binding sites. It includes a two-layer CNN and GCN in parallel to extract the hidden features, followed by a fully connected layer to make the prediction. DeepPN discriminates the RBP binding sites on learnable representation of RNA sequences, which only uses the sequence data without using other data, for example the secondary or tertiary structure data of RNA. DeepPN is evaluated on 24 datasets of RBPs binding sites with other state-of-the-art methods. The results show that the performance of DeepPN is comparable to the published methods.

**Conclusion:**

The experimental results show that DeepPN can effectively capture potential hidden features in RBPs and use these features for effective prediction of binding sites.

## Introduction

RNA-binding proteins (RBPs) are highly involved in cellular processes contributing to gene regulation [1, 2], for example RNA editing, mRNA localization and translational regulation [3]. Detecting the binding sites of RBPs has become an important research objective [4]. However, those approaches of analysis and prediction of RBP binding sites are often time-intensive and subject to experimental variation. The experimental approaches for RBPs site detecting include high-throughput sequencing of RNA isolated by crosslinking immunoprecipitation (HITS-CLIP) which is a method for genome-wide of RNA-binding sites or RNA modification sites in vivo [5], light-activated ribonucleotide enhanced cross-linking and immunoprecipitation (PAR-CLIP) which is a biochemical method used to detect sites of protein-mRNA interaction sites [6] and individual-nucleotide resolution cross-linking and immunoprecipitation (iCLIP) which can identify RNA-protein binding sites with nucleotide resolution [7]. Those methods not only bring the richness of sequencing, but also increase the complexity of biological experiments.

Considering the limitation of experimental methods, many computational tools have been developed to improve the detecting of RBPs binding sites. MEMERIS [8] detects the RBPs binding information with the help of simultaneously integrating information about secondary structures and sequences. RNAcommender [9] uses matrix factorization methods to infer binding RNAs for RBPs by employing protein domain composition and the secondary structures of RNA. CapR is an algorithm that calculates the RBPs binding sites with secondary structural context [10]. RNAcontext learns both the sequence and structure binding preferences of RBPs and assumes that the primary role of RNA secondary structure in RBP binding is to establish a structural context for the RNA sequence recognized by the RBP [11]. The iONMF [12] integrates multiple information to detect the binding sites, such as k-mer sequence data, secondary structure information and Gene Ontology information.

The methods discussed above are mainly based on mathematical computation. For example, RNAcommender uses factorization of matrices to construct a model. QRS [13] proposes a combination of hierarchical clustering and spectral clustering for scRNA-seq analysis. Recently, with the rapid development of deep learning algorithms, deep learning has gradually become a new research hotspot in computational biology with its ability to detect hidden features in large-scale biological data to make predictions [14]. Given the good results achieved by the Convolutional Neural Network (CNN) [15] for tasks such as image classification, (like the applications to X-ray imaging [16]), CNN are receiving more attention from biologists. After numerous researches such as DeepSEA using CNN to identify functional effects of noncoding variants [17] and Basset which offers a powerful computational approach to annotate and interpret the noncoding genome by applying the CNN [18], the CNN has been the main method to capture the RBPs information in various deep learning methods. For example, CNN is applied in DeepBind to improve the performance of detecting the RBPs binding sites [19]. Meanwhile, CNN has also been combined with other deep learning methods. The iDeep utilized the deep belief networks (DBNs) and CNNs to predict the RBPs binding sites [20]. DeeperBind concatenated a long short-term memory network (LSTM) [21] layer based on the original DeepBind method [22]. iDeepS constructed two separate CNNs to learn the sequence data and secondary structural information of RNA respectively. It used the LSTM after CNN to help infer the binding sites [23]. DanQ dealt with the long-term dependencies in the output of CNNs by using the LSTM [24]. Some methods do not use CNN, like Deepnet-rbp, who utilized the DBNs to calculate both sequence and tertiary structural information of RBPs [25].

Another rapidly developed deep learning method, especially in recent years, is Graph Neural Networks (GNNs). GNNs are learnable methods used to detect the hidden feature of non-Euclidean when it come up. The Graph Convolutional Network (GCN) is the most predominant one. GCNs are mainly divided into two categories, spectral based and spatial based GCNs [26]. It was first proposed by Bruna et al. in 2013 [27] based on spectral theory and convolution theorem. Considering its high time complexity, many researchers make efforts to improve it [28, 29]. In spectral based GCN methods, the most popular one is the Chebyshev method, which is used to construct the ChebNet [28]. With the Chebyshev approximation, it could be efficiently computed by applying fast Fourier transforms. Graph Attention Network (GAT) is a well-known spatial based GCN method [30]. GCNs have become popular in protein analysis, drug discovery [31] and medical research. In Decagon [32], the GCN is utilized in embedding the multi-modal graphs of drugs to predict drug combinations on side effects. The standard molecular feature extraction methods are generalized based on circular fingerprints by using the GCN [33]. It has also been applied in the research of protein structure and crystal property prediction [34, 35]. On the multi-modal functional magnetic resonance imaging (fMRI) issues, Qu et al. [36] proposed a deep learning model based on multi-modal GCN for multiple data fusion in 2021. The model captured both the hidden features of fMRI on time series and the function of brain regions. Good results were achieved on wide range achievement test. Most researches apply GCN on non-Euclidean data, however, GCN could also deal with Euclidean data as well, such as image semantic segmentation [37]. In this paper, the ChebNet which is one of the spectral GCNs is used to construct the DeepPN method.

In this work, we propose a parallel deep neural network named as DeepPN that is based on CNN and ChebNet, and apply it to identify RBPs binding sites on 24 real datasets. The feature vectors are fused by the convolutional neural network and the graph convolutional neural network. In DeepPN, the CNN module and ChebNet module are in parallel, which means they extract the hidden features at the same time from the learnable representations of RNA sequences. After CNN and ChebNet capturing the features from the RNA sequences, there is a concatenate layer utilized to combine the feature vectors from two neural networks and then input them to the fully connected layers for the prediction. The network is evaluated on 24 datasets from GraphProt [38]. Experimental results show that our method achieves competitive results with other published methods, and can extract more discriminative features from RNA sequences on some datasets than existing methods.

## Related work

RNA-binding proteins have always played a significant role in the study of gene regulation and it is also an important pathway for gene related researches. For instance, Embryonic lethal abnormal vision protein 1 (embryonic abnormal vision like 1/human antigen R, ELAVL1/HuR) is an RNA-binding protein involved in differentiation and stress response, mainly through stabilizing messenger RNA (mRNA) [39]. It has been shown that ELAVL1 protein can promote tumor cell proliferation through binding to a series of proliferation-related target mRNAs and through post-transcriptional regulation, leading to increased expression of target mRNAs involved in cell cycle progression and cell division. The gradual application of high-throughput sequencing technologies has led to a deeper exploration of previously understudied biodiversity, which encompasses different scientific fields such as protein binding site prediction, resulting in the accumulation of a large amount of biological data. Although these traditional research tools based on biological experiments can be effective in these areas, they are overwhelmed by the massive output of biological data. They are often impractical or too expensive when dealing with such large and complex data. All of these have greatly contributed to the development of deep learning technology in the field of bioinformatics represented by RNA-binding proteins.

Deep learning is a data-oriented research method with matrix data. This allows deep learning to have better analytical performance in the face of sparse matrices of high dimensionality. Bioinformatics data are often highly sparse matrix, such as DNA or RNA sequence data stored in one-hot form, which makes deep learning algorithms aptly exploit their ability to find hidden features in high-dimensional data and achieve better analysis results. For example, in Zeng et al. [40], they applied a systematic study of CNNs models for DNA sequences. This study shows that multiple filters enhance network learning of sequence data by targeting the effects of different width, depth, and pooling layer designs in CNN on the analysis of sequence and motif of DNA-binding proteins. In a recent study by Zhen et al. [41], a deep learning model consisting of LSTM with attention mechanism [42] was proposed for analyzing the RBP binding sites after cutting using k-mer method for RBP sequences. This method tests the effect of varying the length of the k-mer vector on the model performance. The effect of performance is demonstrated by showing the variation of model performance under various k-mer related parameter settings.

With the continuous development of graph convolution, GCN has been gradually applied to the field of bioinformatics. Xuan et al. [43] proposed a deep learning model based on GCN and CNN for lncRNAs in 2019. The framework has two parallel dual branches, one of which is a GCN branch for topological information of lncRNAs and associated diseases. The other branch uses CNN to analyze local features. However, in our experiments, we use the GCN directly to capture hidden features within the sequence, complementing the features captured by the GCN with the CNN in different feature spaces, and do not need to supplement the data external to the sequence. Experimentally, the GCN and CNN form a two-branch structure that yields objective prediction results. This paper validates the effectiveness of applying GCN to RNA sequence analysis.

## Method

### Overview of the DeepPN

To address the problems in existing methods, we have proposed a parallel deep neural network DeepPN. Generally speaking, a too deep structure would cause the sequence features captured early to gradually disappear as the depth deepens. Therefore, in this paper, DeepPN tries to improve the feature capturing capability for RBP sequences from the width perspective in a parallel way. Meanwhile, it is expected that the methods under different perspectives can capture features that are not the same as each other, so that the captured features can complement each other.

**Fig. 1:**
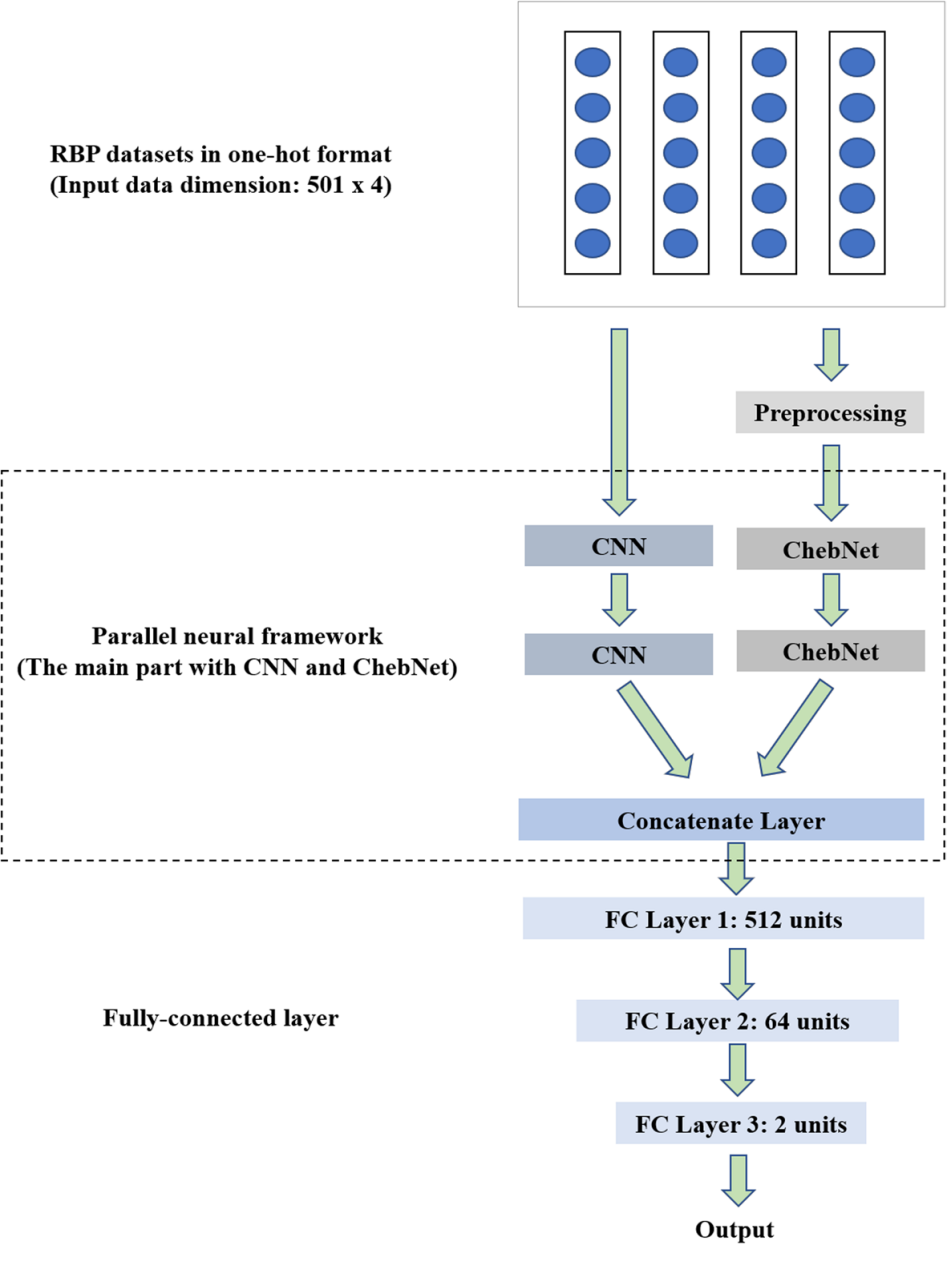
The structure of the DeepPN. The RBP sequence is processed by one-hot method. Then it enters the main part for hidden feature extraction and finally the result is obtained by three fully connected layers

The entire structure of DeepPN is shown in Fig. 1. It has mainly two branches, one is the CNN module and the other is the ChebNet module. The RNA sequences are computed with the following formula:

1 $$H = g_{DeepPN} \left( {g_{CNN} \left( X \right), g_{ChebNet} \left( X \right)} \right)$$

The convolutional module scans the RNA sequences with 4 channels in one-hot format. Concurrently, the ChebNet module works on the same RNA sequences after preprocessing layer. The output of two modules will be concatenated to become a new feature vector. Finally, fully connected layers work with the dropout method [44] as a predictor to generate the probability from the input feature vector. The following formula is utilized to calculate the conditional likelihood:

2 $$\zeta = - \mathop \sum \limits_{i = 1}^{n} y_{i} \log \left( {W \cdot {\text{H}} + b} \right) + \left( {1 - y_{i} } \right)\log \left( {1 - \left( {W \cdot {\text{H}} + b} \right)} \right)$$

where $y_{i}$ represents the truth label. $W$ and $b$ are parameters of the output from the module. $H$ is the high dimensional feature vector of the RNA sequence, which is captured by the method. At last, *n* is the number of the RNA sequences. In order to achieve better performance, the framework will be trained to minimize the value of ${\upzeta }$.

In the training process, the batch size of DeepPN is 16. In the design of the batch size, DeepPN adopts the method of mini batch to reduce the memory load pressure, and at the same time, a reasonable design of mini batch size can speed up the learning efficiency to a certain extent and reach the optimal solution faster. In the selection of the optimizer, DeepPN adopts the Adam method. The learning rate is set to 0.001. Above is an overview of the main structure of DeepPN, in the next section, each part of the ChebNet and CNN modules will be introduced step by step.

### ChebNet in the DeepPN

ChebNet [28] is based on the GCN that is defined in spectral domain [27, 28], which is mainly reflected in different definitions of filters. The filter of the spectral domain GCN could be computed by $g_{\theta } \left( \Lambda \right) = \mathop \sum \nolimits_{k = 0}^{K - 1} \theta_{k} \Lambda^{k}$. ${{\varvec{\Lambda}}}$ is the diagonal matrix of eigenvalues and ${\varvec{\theta}}$ is a vector of polynomial coefficients. However, such filter method still has high computational cost for operations of Fourier basis. To solve this issue, an efficient solution called Chebyshev expansion [28] with approximate kernels is used to improve the filter method. The Chebyshev polynomial $T_{k} \left( x \right)$ is calculated by:

3 $$T_{k} \left( x \right) = 2xT_{k - 1} \left( x \right) - T_{k - 2} \left( x \right)$$

With $T_{0} = 1$ and $T_{1} = x$, Formula (3) is capable to reduce computational complexity significantly due to its recursion. The filter can be redefined as:

4 $$g_{\theta = } \mathop \sum \limits_{k = 0}^{K - 1} \theta_{k} T_{k} \left( {{\tilde{\mathbf{\Lambda }}}} \right),{\text{where}}\;{\tilde{\mathbf{\Lambda }}} = 2{{\varvec{\Lambda}}}/\lambda_{max} - I_{n}$$

In this paper, there is a two-layer ChebNet used to capture the features from the RNA sequences, which is shown in Fig. 1. We assume that the hidden features exist in the structure of the RBPs sequences. Therefore, a two-layer ChebNet with activation method and flatten method is designed. The raw data will be preprocessed before they are measured by two ChebNet layers. The output of this module can be represented as the following formula:

5 $$v_{j} = F_{Flatten} \left( {Leaky{\text{Re}} LU\left( {g_{ChebNet} \left( {l\left( X \right), Adjacency\left( X \right)} \right)} \right)} \right)$$

The $g_{ChebNet}$ means the function of the ChebNet and $l\left( x \right)$ means raw data after preprocessing layer. ChebNet also needs a filter, and the most common way is to rely on the adjacency matrix of the data. To avoid over-smoothing, in this module, the activation function uses LeakyReLU [45] method. Once the data enters ChebNet module, the information is aggregated. After the flatten function, $v_{j}$, the final vector with high-dimensional feature information, will be generated.

### CNN in the DeepPN

In DeepPN, a two-layer convolutional neural network is adopted to calculate the local hidden information of the RNA sequences. There are convolutional operation, activation function and flatten operation in the convolutional module. Each convolutional layer has the same kernel size. Moreover, the first layer contains 16 filters and second layer has 32 filters. The second layer will be fed feature representation from the first layer. In each convolutional filter, a sliding window with kernel size is used to calculate the local hidden features of the RNA sequences. The output of the convolutional module can be represented as the following formulas:

6 $$\left\{ {\begin{array}{l} {v_{j} = F_{Flatten} \left( {Leaky{\text{Re}} LU\left( {f^{\prime}\left( X \right)} \right)} \right)} \\ {f^{\prime}\left( X \right) = W^{f} \cdot \left( X \right) + b^{\prime} } \\ \end{array} } \right.$$

In Formula (6), $F_{Flatten}$ represents the flatten operation, and $f^{\prime}$ denotes the convolution operation. The LeakyReLU is for the activation operation. $X$ is the input RNA sequences which are operated by the one-hot method. $W^{f}$ represents the sliding window with kernel size. $b$ is the bias term. Vector $v_{j}$ is calculated by the filter that includes the activation, convolutional and flatten operation.

## Results

Our method was built with Keras in python and the hardware is the NVIDIA Quadro RTX5000. The RAM of GPU is 16 gigabytes and the hard drive storage space is 2 terabytes. To evaluate the performance of DeepPN, the accuracy and loss of the prediction of RBPs binding sites are measured on the test dataset, which are also compared with other state-of-the-art methods on the same RBP dataset. The data analysis process is as follows: firstly, CNN and ChebNet capture the hidden features, then fuse the features, and finally the fully-connected layer performs classification and prediction. The whole experimental procedure is that the data is pre-processed before the analysis is performed, making the data fit the model analysis requirements. Subsequently, data analysis is performed to obtain data analysis results, which contain metrics such as accuracy. These metrics are then analyzed and compared to produce the experimental results.

### RBPs binding sites datasets

Our experiments are evaluated on 24 datasets which are RBPs binding sits from the HITS-CLIP, PAR-CLIP and iCLIP methods. The positive RBPs binding sites data in 23 datasets are obtained from doRiNA [46] except the PTB binding sites dataset is from the research of genome-wide analysis of PTB-RNA [47]. Each dataset has positive and negative RBPs binding sites data, in which the positive data are from the CLIP-based experiment results and the negative results are created by using bedtools shuffling the coordinates of binding sites within all genes with at least one binding site [48]. Bedtools is a software used for the comparison, manipulation and annotation of genomic features data.

In generating data that can be learned for training, kDeepBind [49] proposes a method to generate sequence feature vectors in a k-gram [50] statistical way to assist in the analysis. The k-gram method counted the frequencies of different length permutations of four bases, A, G, C and U, occurring in the sequences. The following feature extraction formula is satisfied when k is 3:

7 $$\begin{aligned} S & = S_{1} \cup S_{2} \cup S_{3} = \left\{ {N_{i} } \right\} \cup \left\{ {N_{i} ,N_{j} } \right\} \cup \left\{ {N_{i} ,N_{j} ,N_{k} } \right\} \\ & = \left\{ {{\text{A}},\;{\text{G}},\;{\text{C}},\;{\text{U}},\;{\text{AA}},\;{\text{AC}}, \ldots ,{\text{GG}},\;{\text{AAA}},\;{\text{AAC}}, \ldots ,\;{\text{GGG}}} \right\} \\ \end{aligned}$$

in which $S$ is the overall ensemble and $N_{i}$, $N_{j}$ and ${ }N_{k}$ denote the permutations of different A, G, C and U.

Considering the methods of processing data, the time for three types of data processing methods, one-hot, k-gram and k-mer, were compared, as shown in Table 1. For this comparison, the 101 length in kDeepBind [49] was used as the standard intercepted sequence length. Also, with k-gram, k was taken as 3. In k-mer, k was set to 4. From Table 1, it can be demonstrated that one-hot is the fastest in processing data. Although the k-gram method can obtain the statistical features of the sequences, it is time-consuming. Moreover, when the input of different length of sequences need to be filled with placeholders, the complexity of the k-gram method increases steeply and it is not a very efficient way to process the data. In this paper, it is preferred that the model capture the hidden features by itself thus reducing the complexity of the preprocessing work. Therefore, only one-hot approach is used for training prediction data generation in the experiments.

**Table 1:** Time spent in processing data for one-hot, k-gram and k-mer (seconds)

RBPs	One-hot(s)	k-gram(s)	k-mer(s)
C17ORF85 PAR-CLIP	1.21	166.94	56.26
CAPRIN1 PAR-CLIP	4.43	698.55	236.86
C22ORF28 PAR-CLIP	5.17	776.85	264.13
ALKBH5 PAR-CLIP	0.72	108.97	36.60
ELAVL1 HITS-CLIP	4.86	730.46	247.91
HNRNPC iCLIP	10.34	1707.43	544.39
SFRS1 HITS-CLIP	9.56	1646.51	557.26
AGO2 HITS-CLIP	21.79	3801.02	1310.53
TDP43 iCLIP	39.82	6768.75	2195.09
AGO1-4 PAR-CLIP	17.09	2875.98	975.49
TIAL1 iCLIP	21.40	3501.71	1181.97
TIA1 iCLIP	7.76	1350.94	454.44
EWSR1 PAR-CLIP	8.70	1373.17	467.09
ELAVL1 PAR-CLIP(A)	11.60	2005.25	677.86
ELAVL1 PAR-CLIP(B)	5.19	847.13	288.93
FUS PAR-CLIP	18.47	2863.57	994.37
PUM2 PAR-CLIP	5.65	904.35	307.65
IGF2BP1-3 PAR-CLIP	4.61	696.19	234.87
MOV10 PAR-CLIP	7.79	1214.91	429.22
ELAVL1 PAR-CLIP(C)	58.01	9836.02	3327.17
ZC3H7B PAR-CLIP	12.16	1955.89	656.31
PTB HITS-CLIP	24.31	3705.75	1285.36
TAF15 PAR-CLIP	4.78	702.27	237.64
QKI PAR-CLIP	6.17	982.99	328.93
Sum	**311.58**	51221.59	17296.34
Average	**12.98**	2134.23	720.68

The shortest total time and average time among the three data processing methods are shown in bold font

The length of sequence of RBPs binding sites is set the same as iDeepV [51]. After they are processed by the one-hot encoding method, they will form a matrix of positive and negative samples that can be accessed by the model and applied for model training. The total number of samples are shown in Table 2. Here we randomly divide each dataset into training set and test set with a ratio of 8:2. AGO1-4 PAR-CLIP is an assembled dataset which combines the data from AGO1 PAR-CLIP to AGO4 PAR-CLIP. Similarly, IGF2BP1-3 PAR-CLIP integrates the datasets from IGF2BP1 PAR-CLIP to GIF2BP3 PAR-CLIP. The ELVAL1 HITS-CLIP, ELAVL1-CLIP(A), ELAVL1 PAR-CLIP(B) and ELAVL1 PAR-CLIP(C) all contain ELAVL1 binding sites derived by different experiment techniques.

**Table 2:** The number of total samples including positive and negative samples in each dataset

RBP	Samples	RBP	Samples
C17ORF85 PAR-CLIP	3754	EWSR1 PAR-CLIP	31649
CAPRIN1 PAR-CLIP	16041	ELAVL1 PAR-CLIP(A)	51249
C22ORF28 PAR-CLIP	18505	ELAVL1 PAR-CLIP(B)	18702
ALKBH5 PAR-CLIP	2410	FUS PAR-CLIP	66061
ELAVL1 HITS-CLIP	17031	PUM2 PAR-CLIP	17343
HNRNPC iCLIP	41266	IGF2BP1-3 PAR-CLIP	15377
SFRS1 HITS-CLIP	36633	MOV10 PAR-CLIP	26780
AGO2 HITS-CLIP	92346	ELAVL1 PAR-CLIP(C)	238888
TDP43 iCLIP	167110	ZC3H7B PAR-CLIP	40980
AGO1-4 PAR-CLIP	68212	PTB HITS-CLIP	88274
TIAL1 iCLIP	78984	TAF15 PAR-CLIP	13904
TIA1 iCLIP	34184	QKI PAR-CLIP	19418

### Performance of the DeepPN

**Fig. 2:**
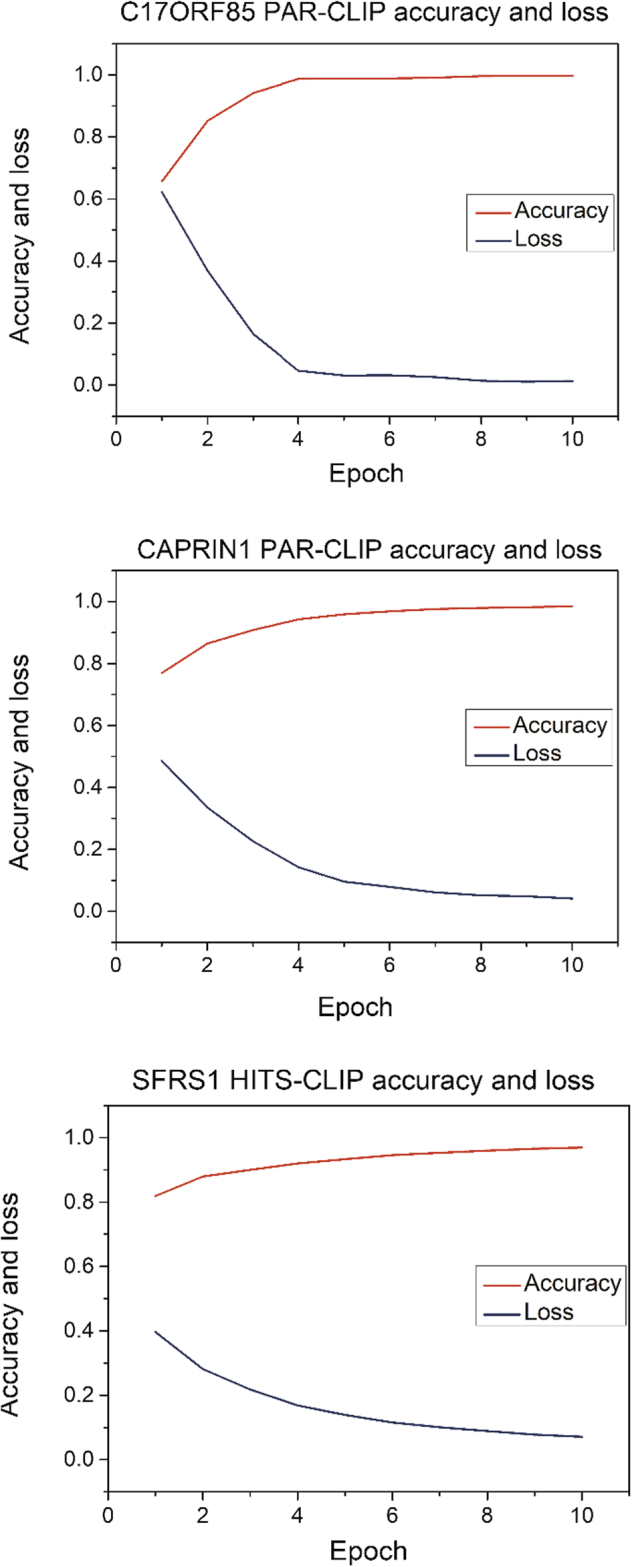
The accuracy and loss without using early-stopping method in C17ORF85 PAR-CLIP, CAPRIN1 PAR-CLIP and SFRS1 HITS-CLIP

Considering that different RBP datasets have different amounts of data, the same hyperparameters may have different training effects in the face of different amounts of data, for example, the training processes of CAPRIN1 PAR-CLIP, C17ORF85 PAR-CLIP and SFPS1 HITS-CLIP are different as shown in Fig. 2. The accuracy in test datasets on SFRS1 HITS-CLIP and CAPRIN1 PAR-CLIP are both over 0.8, i.e., 0.8485 and 0.8308. In this experiment, DeepPN is required to avoid the problem of overfitting when facing data with different scales, in order to achieve better performance as much as possible. The problem of overfitting is that as the model is continuously trained, invalid data may be added to the learning as potential features due to the requirement to continuously improve the results, thus resulting in a situation where the actual prediction results keep decreasing. Therefore, this situation needs to be avoided as much as possible. Based on the above idea, a method called early-stopping is adopted in our experiment, which is designed to monitor the performance of the model. When the performance of the model tends to decrease according to the metrics which generally is the correctness rate, further training of the model is stopped at a reasonable point in time. The patience of the early stopping for model performance decreasing is set up as 2 epochs on the accuracy of the test dataset, which means that if the accuracy of the test dataset decreases in 2 epochs, the model stops the prediction and outputs the result.

**Fig. 3:**
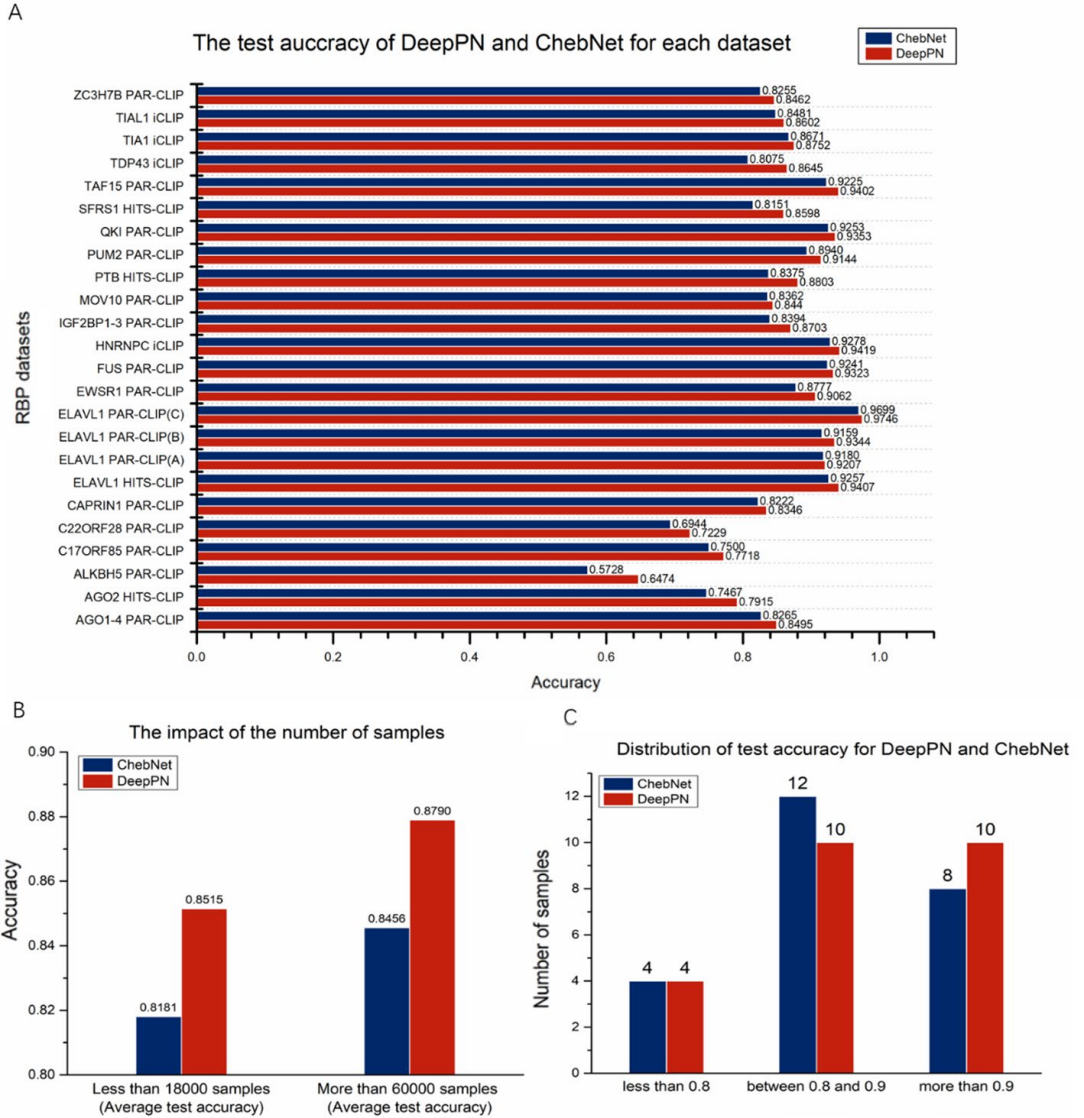
The test accuracy on all the RBP datasets for DeepPN and ChebNet (**A).** The test accuracy on large datasets are much better than small datasets for both DeepPN and ChebNet (**B**). The distribution of the test accuracy for DeepPN and ChebNet (**C**)

The accuracy results incorporating the early stopping method is shown in Fig. 3, part A. In this experiment, the comparison results with ChebNet is added. From the figure, it can be found that the prediction results are inferior to DeepPN when only relying on ChebNet which is a GCN algorithm. Compared to the results of ChebNet, most datasets achieve acceptable results. The ELAVL1 PAR-CLIP(C) has the best performance among all the datasets, which is 0.9746. For both methods, the more samples used for training and testing, the better results are likely to be obtained. The relationship between sample numbers and the test accuracy is illustrated in in Fig. 3, part B. The average test accuracy in 7 datasets with more than 60,000 samples and 7 datasets with less than 18,000 samples are chosen to be compared, and it can be seen that the larger the sample size, the better the results achieved. However, in both categories of datasets, DeepPN achieves better results than ChebNet alone. The ALKBH5 PAR-CLIP gets the lowest accuracy 0.6474 for both methods. That may be because it is the dataset with lowest number of samples, which may limit the number of features detected and affect the accuracy of prediction. There are 10 datasets whose accuracy results are exceeded 0.9 and 20 datasets whose accuracy are exceeded 0.8 for both methods in Fig. 3, part C. Nevertheless, DeepPN has 10 datasets above 0.9 compared to 8 for ChebNet, and DeepPN outperforms ChebNet.

### Comparison with other methods

To compare DeepPN with other related work, we have realized three typical methods as baselines, including GraphProt, Deepnet-rbp and iDeepV. These four methods include deep learning method, non-deep learning method, a method based on structural data, and deep learning method with k-mer function. The results are shown in Table 3. The AUC (Area Under Curve) is used to evaluate those four methods.

**Table 3:** The AUC results for each method

RBP	DeepPN	GraphProt	Deepnet-rbp	iDeepV
C17ORF85 PAR-CLIP	**0.837**	0.800	0.820	0.740
CAPRIN1 PAR-CLIP	**0.886**	0.855	0.834	0.824
C22ORF28 PAR-CLIP	0.785	0.751	0.792	**0.823**
ALKBH5 PAR-CLIP	0.660	0.680	**0.714**	0.643
ELAVL1 HITS-CLIP	**0.978**	0.955	0.966	0.966
HNRNPC iCLIP	0.977	0.952	0.962	**0.979**
SFRS1 HITS-CLIP	**0.936**	0.898	0.931	0.905
AGO2 HITS-CLIP	0.868	0.765	0.809	**0.886**
TDP43 iCLIP	**0.936**	0.874	0.876	0.935
AGO1-4 PAR-CLIP	0.912	0.895	0.881	**0.925**
TIAL1 iCLIP	0.926	0.833	0.870	**0.929**
TIA1 iCLIP	0.928	0.861	0.891	**0.941**
EWSR1 PAR-CLIP	0.954	0.935	**0.966**	0.962
ELAVL1 PAR-CLIP(A)	0.967	0.959	0.966	**0.973**
ELAVL1 PAR-CLIP(B)	**0.976**	0.935	0.961	0.962
FUS PAR-CLIP	0.977	0.968	**0.980**	0.976
PUM2 PAR-CLIP	0.952	0.954	**0.971**	0.965
IGF2BP1-3 PAR-CLIP	**0.928**	0.889	0.879	0.923
MOV10 PAR-CLIP	**0.904**	0.863	0.854	0.896
ELAVL1 PAR-CLIP(C)	**0.994**	0.991	0.994	0.990
ZC3H7B PAR-CLIP	**0.898**	0.820	0.796	0.883
PTB HITS-CLIP	0.938	0.937	**0.983**	0.936
TAF15 PAR-CLIP	0.974	0.970	**0.983**	0.978
QKI PAR-CLIP	0.975	0.957	**0.983**	0.965
Average	**0.919**	0.887	0.903	0.913

The best performance is marked in bold

The AUC results for GraphProt, Deepnet-RBP and iDeepV are taken from original papers

**Fig. 4:**
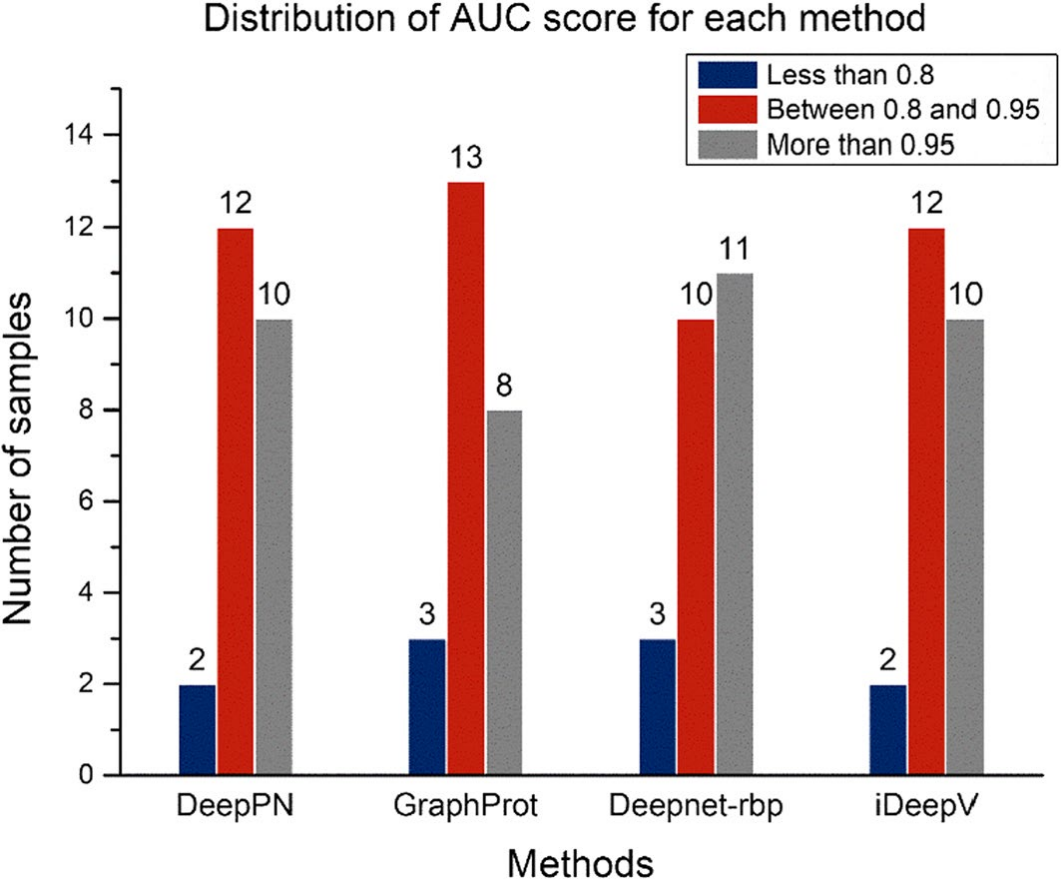
The quantity of different ranges of AUC score for each method

The average AUC of DeepPN, GraphProt, Deepnet-rbp and iDeepV are 0.919, 0.887, 0.903 and 0.913. The data distribution of AUC score for the DeepPN is similar to that of iDeepV. The reason of this similarity may lie in the fact that DeepPN and iDeepV are two methods without using the structural data and only using the sequence data. However, the result of the DeepPN is slightly better than that of the iDeepV on average AUC score. The performance of the 4 methods is shown in Fig. 4. DeepPN and iDeepV show same number of exceed AUC scores for more than 0.8 on 24 datasets, outperforming Graphport and Deepnet-rbp.

DeepPN returns the highest AUC results for 10 of the RBPs datasets, while Deepnet-rbp returns the highest AUC for 8 RBPs datasets including identical AUC socre for the ELAVL1 PAR-CLIP(C). iDeepV ranks the third with highest AUC in 7 RBPs datasets. Graphprot, not a deep learning method, does not return the highest AUC for a dataset.

Specifically, first as both use only sequence data, DeepPN classifies better than iDeepV on some datasets. For instance, C17ORF85 PAR-CLIP has a result of 0.873 on DeepPN and 0.740 on iDeepV,. a 13% improvement relative to iDeepV. DeepPN still outperforms Deepnet-rbp which uses structural data. The AUC result for the ZC3H7B PAR-CLIP dataset from DeepPN is 0.898, which is a 12% improvement relative to Deepnet-rbp. Similarly, in TDP43 iCLiP, DeepPN improves by 6.8% and in IGF2BP1-3 PAR-CLIP improves by 5.5%.

Also, it can be observed that for DeepPN and iDeepV, often the larger the dataset is, the better classification results are achieved. For example, as the ELAVL PAR-CLIP (C) dataset with the largest data volume, DeepPN achieved the best AUC result of 0.994. The second largest TDP43 iCLIP achieved the best result relative to the other three methods. For the smaller datasets, the performance of all methods decreases. However, compared with the iDeepV, our method performs better on some smaller datasets. In C17ORF85 PAR-CLIP dataset with 3754 samples, DeepPN outperforms the other methods. Conversely, in the very small ALKBH5 PAR-CLIP which only has 2410 samples, Deepnet-rbp and GraphProt show better performance than DeepPN; notably, this is the only dataset where GraphProt convincingly outperforms DeepPN (AUC 0.680 vs 0.660).

## Discussion

DeepPN is presented in this paper which focuses on the prediction of RBPs binding sites from sequence information alone. It is aimed to assist researchers to prioritize candidate RBPs binding sites rather than using high-cost, time-consuming experimental investigations including genome wide CLIP-seq methods and functional testing in vivo and in vitro model system.

Meanwhile, it is found that a larger data volume is more helpful for the model to achieve better prediction results when using only sequence data for training. Throughout the performance results of DeepPN and iDeepV, both achieve good results on the dataset with larger data volume represented by ELAVL1 PAR-CLIP(C), but the results on ALKBH5 PAR-CLIP with smaller data volume are more average. It may be indicated that larger data contain richer hidden features, making it easier for the model to capture the features. Correspondingly, in the case of smaller sequences, the additional structural information helps to improve the classification results, which is reflected by the best results of Deepnet-RBP on ALKBH5 PAR-CLIP.

In this experiment, DeepPN differs from kDeepBind [49] and iCircRBP-DHN [52] in utilizing statistical frequencies to complement the features. It is built with more focus on enhancing the analysis with different deep learning models. At the same time, it does not focus too much on the processing of the dataset itself, while in the recent study, EDCNN [53] is based on iDeepE [54], and the data is cut into a local analysis part and a global analysis part to make the analysis effect improved. In future research, we will track the partitioning of the dataset and enhance the interpretability of the model.

## Conclusion

In this paper, a deep parallel method called DeepPN is proposed with CNN and ChebNet for the RBP binding sites prediction. Moreover, the ChebNet based on the spectral GCN has been utilized in the RNA sequence analysis, which indicates that GCNs are beneficial to capture relative features from RNA sequences. The proposed method is evaluated on 24 datasets with RBPs. Considering that GCNs are mostly used in protein analysis now, our work suggests that GCN can also be used in sequence data analysis.

## Data Availability

The dataset of RNA-binding protein binding sites can be download form http://www.bioinf.uni-freiburg.de/Software/GraphProt/. The code used or analyzed during this study are available from the corresponding author on reasonable requests.

## Abbreviations

CNN: Convolutional Neural Network
GCN: Graph Convolutional Network
RBP: RNA-binding protein
ChebNet: Chebyshev spectral convolutional neural network
AUC: Area under the ROC curve
